# Routes of precursors’ migration in remyelination

**DOI:** 10.1093/brain/awaf441

**Published:** 2025-11-20

**Authors:** Majid Ghareghani, Samira Ghorbani

**Affiliations:** Department of Biology, University of Toronto Mississauga, Mississauga, ON L5L 1C6 Canada; Department of Immunology, University of Toronto, Toronto, ON M5S 1A8, Canada; Department of Cell & Systems Biology, University of Toronto, Toronto, ON M5S 3G5, Canada; Department of Biology, University of Toronto Mississauga, Mississauga, ON L5L 1C6 Canada; Department of Immunology, University of Toronto, Toronto, ON M5S 1A8, Canada; Department of Cell & Systems Biology, University of Toronto, Toronto, ON M5S 3G5, Canada

**Keywords:** neural stem cells, remyelination, migration, oligodendrocyte precursors, signaling pathways, subventricular zone

## Abstract

Remyelination, the biological process of restoring myelin sheaths, is critically dependent on the successful recruitment of oligodendrocyte lineage cells. However, this process involves at least two functionally distinct endogenous reservoirs: the widely distributed parenchymal oligodendrocyte precursor cells (pOPCs) and neural stem cells (NSCs) originating from the subventricular zone (SVZ). Emerging evidence reveals that these populations employ different migratory strategies. The pOPC response is characterized by rapid local proliferation but severely constrained, short-range migration, often limited by an inhibitory lesion microenvironment. In contrast, SVZ-derived precursors, including fate-switching neuroblasts, are capable of long-range, adaptive migrations, navigating complex routes by co-opting both vascular and parenchymal scaffolds.

This review synthesizes these divergent phenomena into a unifying model of competing migratory programmes. We posit that the success or failure of endogenous repair depends on a dynamic interplay between the limited local pOPC response and the recruitment of the more plastic, long-range SVZ-derived cohort. We examine how the oligovascular niche and pivotal signalling cascades, including Wingless/Int-1–β-catenin (Wnt/β-catenin), bone morphogenetic protein (BMP), and sonic hedgehog/glioma-associated oncogene (Shh/Gli), function as critical regulators in this process, dictating which migratory strategy predominates in a given pathological context.

Viewing remyelination as a dynamic balance between these two cellular systems helps explain why this repair process often fails. Ultimately, understanding these migratory programmes as an interconnected system, rather than as isolated components, is essential for developing more effective interventions that promote functional myelin repair in demyelinating diseases.

## Teaser

Ghareghani and Ghorbani show that the brain relies on two distinct cell types—parenchymal oligodendrocyte precursor cells and neural stem cells from the subventricular zone—to repair myelin damage. Each follows different migratory routes, and effective therapies must support both pathways to enhance myelin repair.

## Introduction

The restoration of myelin sheaths to denuded axons, a process known as remyelination, represents a critical objective in the development of therapeutic strategies for demyelinating pathologies such as multiple sclerosis (MS). This endogenous repair mechanism fundamentally relies on the orchestrated recruitment and functional maturation of cells capable of differentiating into new, myelin-producing oligodendrocytes.^1-4^ The adult CNS harbours a population of oligodendrocyte precursor cells (OPCs) that possess an intrinsic capacity for remyelination. In this paper, we distinguish between two main populations of OPCs: the resident parenchymal cells (pOPCs) and those derived from the subventricular zone (SVZ-derived OPCs). However, the efficacy of these pOPCs often diminishes in the context of chronic or severe demyelinating insults, necessitating the exploration of alternative cellular sources for myelin repair. Neural stem cells (NSCs), which persist in specialized neurogenic niches of the adult brain, have received considerable attention in this regard. These cells, largely quiescent under physiological conditions, retain the remarkable potential to differentiate into all three major neural lineages: neurons, astrocytes and oligodendrocytes, thereby presenting an attractive endogenous reservoir for augmenting myelin regeneration.^5^

The principal neurogenic regions in the adult mammalian brain are the SVZ lining the lateral ventricles and the subgranular zone (SGZ) of the dentate gyrus in the hippocampus. The SVZ, in particular, is a complex and highly organized niche. It contains primary NSCs, designated as type B cells, which exhibit astrocytic characteristics. These type B cells give rise to rapidly dividing transit-amplifying progenitors (type C cells), which, in turn, generate migratory neuroblasts (type A cells).^6^ Under normal physiological conditions, these neuroblasts predominantly migrate along the rostral migratory stream (RMS) to replenish interneurons in the olfactory bulb (OB).^6^ However, in response to demyelinating injury, a subpopulation of these NSCs can be activated and diverted from their canonical fate, generating new oligodendrocytes that successfully integrate into damaged white matter tracts and contribute to functional recovery.^5^

Substantial progress has been made in identifying the individual molecules that influence precursor cell behaviour. This is exemplified by a recent review from Cao and colleagues^7^ that comprehensively compiles the molecular factors known to regulate OPC migration during development and repair. This foundational knowledge, however, largely centres on the OPC lineage, which points to a more complex, systems-level question: how are these molecular instructions interpreted and integrated differently by the CNS’s two principal regenerative reservoirs—local pOPCs and niche or SVZ-derived OPCs—to produce divergent migratory behaviours?

This review addresses this question by integrating the current literature to compare the distinct migratory strategies of these two precursor cell populations. We specifically contrast the highly constrained, territorially restricted movement of resident pOPCs with the remarkable, adaptive migrations of the SVZ-NSC lineage, which can involve fate-switching and the co-opting of both vascular and non-vascular routes. By framing the repair process as a dynamic interplay between these two distinct cellular systems, we critically revisit the role of the oligovascular niche, not simply as a passive scaffold or ‘highway’, but as a key regulator of migratory choice and cell fate. Our objective is to describe how the success or failure of remyelination depends on the context-specific dominance of one migratory strategy over another, which, in turn, is regulated by a complex cell-intrinsic programme and powerful microenvironmental cues. A deeper understanding of these multifaceted programmes holds the promise of developing novel therapeutic strategies that can selectively and potently enhance functional myelin repair.

## The SVZ in development and adulthood: rodent-human differences

The SVZ is a lifelong reservoir of NSCs, but its function shifts dramatically from development to adulthood, with notable differences between rodents and humans. During embryonic development, SVZ-NSCs are highly proliferative. They migrate extensively to populate the developing brain and establish its fundamental architecture.^8^ In contrast, the role of the adult SVZ is more restricted. In rodents, adult NSCs are largely quiescent but can be activated by injury.^9^ Their progeny typically migrate in chains along the RMS to supply new neurons to the OB.^10^ In the adult human brain, however, this SVZ-OB pathway is far less active. While NSCs with proliferative potential are still present in the human SVZ,^11^ their output and function shift significantly with age.^12-14^ Unlike rodents, where neuroblasts generated in the SVZ migrate in dense, organized chains along the RMS to the OB, the human RMS lacks this structured stream in adulthood.^14,15^ Instead, only sparse, disorganized neuroblasts can be found, often migrating singly or in small groups. This reduction in migratory neurogenesis is already evident early in life; by approximately 18 months of age, the production and migration of neuroblasts toward the OB decline sharply and become virtually undetectable in adulthood.^16^ This reduction in neurogenesis is consistent with the proportionally smaller OB in humans, which likely has a lower demand for neuronal replenishment.^17^

Despite this reduction in basal SVZ-OB neurogenesis, the adult human SVZ demonstrates significant plasticity and responsiveness to brain injury.^18,19^ Increased proliferation within the SVZ and the appearance of neural progenitors in perilesional areas have been documented following ischaemic insults or traumatic brain injuries in humans.^20^ Specifically, in the context of demyelinating diseases such as MS, evidence points towards mobilization of the SVZ, characterized by increased proliferation and the emergence of cells expressing early oligodendrocytic markers in proximity to lesions. This suggests that OPCs may be recruited from this niche to sites of damage.^18^ Further underscoring this pathological responsiveness, a study focusing on MS patients reported a significant (3.5-fold) increase in NeuN-positive cells, many of which co-expressed immature neuronal markers like PSA-NCAM and Dlx2, within SVZ regions adjacent to demyelinated lesions.^21^ These neuroblast-like cells were also detected within chronic lesions, displaying features of neuronal integration. Complementing these histological findings, recent in vivo imaging data from paediatric and adult MS patients have revealed significant structural alterations in the SVZ, including increased extracellular free water content and signs of astroglial hypertrophy, particularly in progressive disease stages.^22^ These changes point to a dysregulated niche, where cellular recruitment potential may be impaired by inflammatory or degenerative remodelling. Therefore, while its homeostatic contribution to olfactory neurogenesis appears limited in humans, the adult human SVZ remains a dynamic region capable of mobilizing cells for brain repair, potentially contributing to oligodendrogenesis, in demyelinated areas and offering a potential target for therapeutic intervention. Understanding the mechanisms that guide adult NSCs activation and migration in both rodents and humans is therefore crucial for harnessing their therapeutic potential for CNS repair.

## NSC to OPC differentiation: priming for myelin repair

Before discussing the intricate migratory pathways that adult NSCs and their OPC progeny undertake to reach sites of demyelination—a central theme of this review—it is imperative to appreciate the complex array of molecular and cellular events that govern their commitment to the oligodendrocyte lineage. The differentiation of NSCs, from a state of relative pluripotency or lineage restriction, into mature, myelin-synthesizing oligodendrocytes is not simply a passive step before cellular translocation. Instead, it represents a highly dynamic and rigorously regulated developmental cascade that intrinsically dictates their ultimate therapeutic efficacy. Indeed, the successful contribution of migrating cells to myelin repair is unequivocally dependent upon their prior acquisition of appropriate lineage specification and functional maturation; progenitors arriving at a lesion site must be equipped with the requisite molecular machinery to engage effectively in myelinogenesis.

An extensive body of research, some summarized in Supplementary Table 1, has delineated a number of signals that orchestrate this differentiation process, highlighting the critical importance of cell-autonomous factors. Among these, pivotal transcription factors play indispensable roles. For instance, Olig2 has been well demonstrated to be both necessary and sufficient for directing SVZ progenitors towards oligodendroglial fates, while concurrently suppressing neuronal differentiation.^17^ Constitutive expression of Olig2 in NSCs can effectively prevent neurogenesis and initiate their entry into the oligodendroglial lineage.^17^ Further emphasizing the power of intrinsic genetic programmes, overexpression of the transcription factor Zfp488 in adult SVZ NSCs potently drives their differentiation into the oligodendrocyte lineage, which significantly enhances remyelination and functional recovery following demyelinating injury.^19^ This capacity of Zfp488 is conserved across species, as forced expression in human NSCs instigates an almost exclusive commitment to the OPC lineage, largely inhibiting neuronal and astrocytic differentiation. When transplanted into myelin-deficient animal models, these Zfp488-programmed human NSCs contribute effectively to myelination.^19,23^

Beyond these transcription factors, the targeted activation of specific signalling pathways, such as the fibroblast growth factor (FGF)^24^ and sonic hedgehog (Shh)/Gli pathways^25^ and bone morphogenetic protein (BMP) pathways,^26^ among others, can also profoundly influence NSC fate. For example, transiently augmenting fibroblast growth factor receptor 3 (FGFR3) activity in adult NSCs has been shown to effectively redirect these stem cells from a predominantly neurogenic fate towards an oligodendrogenic one, particularly under demyelinating conditions. This intervention causes a significant increase in the production of SVZ-derived OPCs, markedly improving oligodendrocyte regeneration and subsequent remyelination.^24^ A series of studies using Gli1 and Gli2 mutant mice demonstrated that Shh/Gli signalling plays a nuanced role in regulating NSC mobilization and OPC differentiation.^27-29^ For instance, a study by Radecki *et al*.^28^ on Gli1 and Gli2 mutant mice revealed that Shh/Gli signalling finely tuned the mobilization and differentiation of ventral NSCs in the adult brain. Under normal conditions, Gli1 acts as a brake, limiting NSC differentiation; however, when Gli1 is inhibited following demyelination, a robust surge of SVZ-derived OPC formation occurs, significantly enhancing remyelination. Demyelinating injury further induces an upregulation of Gli2 in these cells, and the balance between Gli1 and Gli2 is essential for proper recruitment and terminal differentiation of ventral NSC progeny into myelinating oligodendrocytes. Indeed, combined loss of both transcription factors dramatically reduces the number of SVZ-derived cells that reach and repair lesions, indicating that while Gli1 restrains differentiation under baseline conditions, Gli2 is required to promote and sustain the remyelination process. In addition, Gli2 has been identified as essential for oligodendrogenesis from adult NSCs.^30^ Thus, based on these observations, high levels of Shh upregulate Gli1 to inhibit oligodendrocyte generation while moderate Shh levels promote oligodendrogenesis from NSCs in the adult brain. Although Shh is primarily secreted by neurons,^31^ the vascular and inflammatory environment may regulate the Shh morphogen gradient, altering its availability to NSCs, indirectly affecting how these precursor populations respond to demyelination. These examples—together with those detailed in Supplementary Table 1, including Wnt/β-catenin signalling,^32^ EGF/EGFR signalling,^33^ hepatoma-derived growth factor (HDGF),^34^ circadian cues^35^ and hypothyroid,^36^ among many others—underscore the significant influence of both intrinsic cellular programmes and extrinsic signaling cues in dictating the oligodendrogenic potential of adult NSCs, a critical prerequisite for their contribution to myelin repair.

## Remyelination dynamics: competition between parenchymal and SVZ-derived OPCs

Both pOPCs and SVZ-derived OPCs can contribute to the remyelination process, although their roles, timing of recruitment and relative efficiency can differ. Typically, pOPCs are the first responders to acute demyelinating injury, rapidly proliferating and differentiating into mature oligodendrocytes to ensheath denuded axons. However, NSCs from the SVZ may be recruited, particularly in scenarios where the pOPC-mediated repair is delayed, insufficient or chronically impaired.^37-39^ This interplay between the two precursor pools creates a dynamic situation where NSCs and pOPCs may compete for access to lesions and resources, or alternatively, compensate for each other’s deficiencies. For instance, recent work by Moyon *et al*.^37^ has shed light on this competitive dynamic within the adult corpus callosum. Their findings suggest that pOPCs are typically the principal mediators of remyelination in this region. However, when pOPC differentiation is genetically abrogated by specifically deleting myelin regulatory factor (Myrf) only in resident OPCs, SVZ-derived OPCs are recruited in significantly greater numbers, effectively compensating for the functional loss of pOPCs. Using a dual-reporter fate-mapping system, this study quantified a 1.7-fold increase in SVZ-derived progenitors, which in turn led to a 2-fold increase in the number of mature oligodendrocytes (CC1-positive) derived from the SVZ during the early recovery phase following cuprizone-induced demyelination. This indicates that SVZ-derived OPCs can rapidly differentiate and contribute to repair when the primary pOPC response is compromised.^37^ These findings underscore a competitive interplay, implying that the rapid functional response of local pOPCs is the key limiting factor for the SVZ’s contribution. When this local repair process is genetically blocked, a temporal window of opportunity is created, allowing the more distantly-derived NSC progeny to access the lesion and successfully contribute to remyelination.^37^ This suggests that therapeutic strategies aimed at enhancing NSC activation and recruitment may hold particular promise in conditions where endogenous pOPC function is impaired. This dynamic interplay between competition and compensation likely occurs within a microenvironment regulated by a complex network of vascular cues, including chemokine gradients and growth factors released from the lesion-associated vasculature, though clarifying the direct role of these signals in modulating the competition remains an important area of research.

## Shifting strategies of NSC migration: from developmental patterning to adult remyelination

NSCs and their progeny adapt their migratory strategies to meet the changing physiological demands of the brain. During development, their migration is highly organized, primarily following glial fibres to build the brain’s structure. In contrast, after an injury in the adult brain, their strategy becomes more flexible and adaptive. They switch from using glial guides to using other structures, like blood vessels and powerful chemical signals (chemotaxis), to navigate to the damaged area.

### Dominant vessel-independent NSC migration in development

During the critical postnatal window of CNS development, the SVZ orchestrates the population of nascent white matter tracts through a highly organized, vessel-independent migratory programme. At this stage, SVZ-derived NSCs give rise to waves of glial-restricted progenitors that are temporally regulated and fated to become oligodendrocytes and astrocytes.^40^ A key study using dynamic time-lapse imaging has dissected the divergent migratory behaviours of these early postnatal SVZ populations with remarkable clarity. Suzuki and Goldman^41^ demonstrated that two geographically and mechanistically distinct migratory pathways emerge from the SVZ. The first route is the well-known sideways (tangential) chain migration of neuroblasts within the RMS, but the second, essential for myelination, is a radial egression of glial progenitors.^41^ These cells, destined for the oligodendrocyte lineage, move outward from the ventricular surface, using the radial glial processes as a structural scaffold to invade the overlying corpus callosum, striatum and developing cortex.^41,42^ This gliogenic migration, predicated entirely on glial scaffolding, is very different from vessel-guided paradigms. Studies confirm that these radially migrating progenitors do not associate with the vasculature during their journey; instead, they maintain a fidelity to their glial guides.^41,43^ This strategy allows the SVZ to send different precursors along separate paths to build both neuronal and glial circuits, apparently without needing guidance from blood vessels. This highlights a developmental strategy where distinct, lineage-restricted precursors—like those identified by Luskin and McDermott^44^—are dispatched along separate paths to build neuronal and glial circuits without relying on guidance from blood vessels.

### Vessel-dependent and independent NSC migration in adulthood: responding to demyelination

In the adult brain, NSC migration continues, albeit in a more restricted fashion, primarily from the SVZ along the RMS to the OB.^45^ A foundational study by Nait-Oumesmar and colleagues^46^ demonstrated that following toxin-induced demyelination, progenitor cells within the adult SVZ proliferate and migrate from their niche toward the lesion, where they successfully differentiate into new, myelinating oligodendrocytes. This injury-induced migration is an adaptive process guided by chemotactic gradients. Similar to the CNS response to stroke, where the SDF-1/CXCR4 axis directs SVZ-derived cells to the infarct,^47^ demyelination establishes a chemical trail that mobilizes progenitors for repair. This allows them to navigate the complex adult parenchyma by employing both vessel-dependent strategies, using the reactive vasculature as a physical scaffold, and vessel-independent movement directly through the brain tissue. More recent and detailed analyses have revealed the surprising identity and profound impact of these migrating cells. Delving into the identity of these precursors, work by El Waly *et al*.^48^ revealed that a significant portion of the responding cells are actually neuroblasts—immature neurons destined for the olfactory bulb—that undergo a spontaneous fate conversion. In response to demyelination, these neuroblasts are diverted from the RMS, gradually downregulating their neuronal identity and upregulating an oligodendroglial programme, thereby converting into early-stage OPCs (hereafter referred to as pre-OPCs), which ultimately mature into functional oligodendrocytes. The significance of this SVZ-derived contribution was quantified by Xing and colleagues,^49^ who showed that in regions adjacent to the niche, such as the rostral corpus callosum, the number of new oligodendrocytes generated from the SVZ can significantly outnumber those from resident pOPCs. Furthermore, they found that the myelin sheaths produced by these SVZ-derived cells are structurally superior, being significantly thicker than those produced by pOPCs, which leads to a more complete restoration of axonal function. Crucially, this potent reparative activity is strictly injury-induced, as oligodendrogenesis from the SVZ does not occur in the healthy brain.^29^ where SVZ-derived cells work alongside resident OPCs to enhance overall remyelination, has been observed repeatedly across demyelinated white matter tracts.^37,38,50,51^ These findings collectively highlight a dynamic and highly plastic repair mechanism where demyelination signals not only recruit progenitors from the SVZ but can specifically hijack and reprogramme neuroblasts in transit, turning them into a source of highly effective, myelin-producing cells.

## Parenchymal OPC migration: dispersal and response across the lifespan

Across the lifespan, the migratory behaviour of OPCs shifts from a programme of broad, long-range dispersal to one of localized, rapid repair. During development, these cells use both vessel-dependent and independent pathways to populate the entire CNS. In adulthood, however, they adopt a very different strategy, characterized by a rapid but spatially-contained response to injury that is often limited by local inhibitory cues.

### Vessel-dependent and independent OPC migration in development

During CNS development, OPCs arise from restricted progenitor domains in the ventral neuraxis and subsequently undergo extensive long-distance migration to populate the entire brain and spinal cord. One of the earliest and most striking features of this developmental OPC migration is their close association with the developing CNS vasculature. Tsai *et al*.^52^ provided compelling evidence that developing OPCs use blood vessels as physical scaffolds for their dispersal. These OPCs interact directly with the outer surface of endothelial cells, extending processes along the vessels and exhibiting two distinct migratory modes: a slower ‘crawling’ motion with continuous vessel contact, and a more rapid ‘jumping’ or saltatory movement where they extend a process from one vessel to a neighbouring one before translocating their cell body. The vascular network, rich in extracellular matrix (ECM) proteins and endothelial-derived factors, not only provides a defined pathway but also likely offers a path of least resistance through the densely packed developing neural tissue.

On the other hand, astrocytes, with their strategically positioned endfeet on the vasculature, regulate the termination of perivascular migration for certain precursor populations, ensuring their timely detachment and integration into the parenchyma. Indeed, Su *et al*.^53^ demonstrated that as astrocyte endfeet ensheath blood vessels, their processes physically mediate OPC detachment by directly displacing OPCs from the vessel surface, a process driven by astrocyte-derived repulsive signals, specifically semaphorin 3a and 6a. Consistent with this, in conditions lacking either astrocytes or with disrupted endfeet, OPCs failed to detach and remained stuck to the vasculature. These findings suggest astrocytes actively release OPCs from an inhibitory perivascular niche to allow for maturation.

However, developmental OPC migration is not exclusively vessel-dependent, a concept directly addressed by Liu *et al*.,^3^ who demonstrated that the majority of neonatal OPC migration occurs along radial glial fibres rather than the vasculature. Using brain lipid-binding protein (BLBP) as a specific marker for radial glia, they revealed that even OPCs appearing in close proximity to blood vessels—over 80%—were, in fact, physically separated from the endothelial wall by an intervening layer of BLBP^+^ glial processes. This indicates that radial glial cells provide the primary migratory scaffold, even in perivascular regions. Further supporting this, the migratory dynamics of OPCs, including their speed and directionality, were not significantly different whether they were near or distant from a blood vessel. These results strongly suggest that developing OPCs are not simply ‘vessel-hitchhikers’, but instead primarily rely on the glial scaffold for their long-range dispersal through the developing CNS.

Another notable example is from Lepiemme *et al*.,^54^ who found that early, first-wave ventrally derived oligodendrocyte precursor cells (vOPCs) guide interneuron migration by using contact repulsion to steer them away from blood vessels. This repulsion is powerful enough to override the attraction to vascular Cxcl12. When a vOPC directly contacts an interneuron, it reverses the interneuron's polarity, forcing it away from the vessel and onto its correct path. This is mediated by Sema6a/6b on vOPCs signalling through Plexin A3 on interneurons. Importantly, this function is unique to these first-wave OPCs; their absence causes interneurons to migrate incorrectly and cluster abnormally, disrupting cortical development.

### Evidence of vessel-dependent OPC migration in adulthood: responding to demyelination

The role of blood vessels as migratory scaffolds for adult OPCs is less understood than in development. However, compelling evidence shows their recruitment to the perivascular niche following demyelination. In the adult CNS, pOPCs remain dispersed throughout the parenchyma, constituting a significant proportion of glial cells. Under normal physiological conditions, they exhibit limited migratory activity, primarily extending and retracting their processes to survey their local environment. However, in response to demyelinating injury, these quiescent pOPCs are rapidly activated. Although adult pOPCs do not typically undertake the long-distance migrations seen in development, their association with blood vessels is still crucial. This perivascular interaction remains relevant, but its primary function appears to transition from guiding migration to providing local signals for cell survival and maturation. Studies by De La Fuente *et al*.^2^ and Palhol *et al*.^55^ in adult demyelinated brains identified an increased OPC density around the vasculature but did not assess migration distance. Indeed, an early study using lysolecithin and ethidium-bromide demyelination in the caudal cerebellar peduncles of adult rat showed that OPCs proliferate quickly after injury and accumulate around cortical and spinal microvessels. In pericyte-deficient PDFGb^ret/ret^ mice, which lack ≈80% of capillary pericytes, the number of lesion-recruited PDGFRα⁺ OPCs and the fraction of those in direct vessel contact were unchanged relative to wild-type, demonstrating that pericytes are dispensable for OPC migration and perivascular positioning *in vivo*. The critical function of pericytes instead emerges later: they deposit laminin-α2 (Lama2) into the vascular basement membrane, creating a niche that boosts OPC survival and speeds their differentiation into mature, myelinating oligodendrocytes, thereby accelerating overall remyelination.^2^ Palhol *et al*.^55^ revealed that approximately 17% of oligodendrocytes and their precursors are in direct contact with vascular basement membranes both during normal and remyelinating conditions. The IL-17/CXCL5 signalling axis provides another example of these complex interactions. As shown by Xiao *et al*.,^56^ endothelial CXCL5 expression under pathological conditions, like diet-induced obesity, promotes OPC association with the vasculature. However, this proved to be detrimental, as the sustained recruitment impaired subsequent remyelination by likely trapping the progenitors in a perivascular, non-productive state.

### Vessel independent OPC migration in adulthood

Despite this vascular association, the overall migratory range of adult pOPCs in response to focal demyelination appears remarkably constrained. Using genetic fate mapping in a lysolecithin-induced demyelination model, Hughes and colleagues^57^ uncovered that while OPCs (NG2+ cells) proliferate robustly and extend dynamic processes, their cell bodies move very little—on average only 2.1 µm per day. Their findings indicate that remyelination is driven by a highly localized response where neighbouring OPCs rapidly proliferate and invade vacant territories through self-repulsion, a process confined to a radius of about 50 µm, rather than by long-distance recruitment. Work by Zawadzka *et al*.^58^ similarly revealed that following toxin-induced demyelination, the vast majority of new remyelinating cells originate from precursors located directly within or very near the lesion, reinforcing the idea of a limited migratory capacity. Instead of migrating far, repair is predominantly achieved through the local proliferation and differentiation of nearby cells. They also highlighted that environmental constraints—such as the presence of astrocytes, ECM components, and inhibitory cues—likely restrict OPC mobility. These local barriers help maintain a tightly organized grid of precursors, ensuring that remyelination is achieved primarily through the activation and expansion of nearby cells rather than through recruitment from distant areas.

In the adult CNS, injury not only triggers OPC proliferation but also alters the extracellular landscape.^59^ Keough *et al*.^60^ demonstrated that after demyelinating injury, astrocytes and the resulting astrogliotic scar markedly upregulate chondroitin sulfate proteoglycans (CSPGs), which may create a biochemical barrier by inhibiting OPC process extension and migration. Their work showed that this inhibitory effect can be mitigated by a small molecule inhibitor, fluorosamine, that reduces CSPG synthesis, thereby promoting a more permissive environment for OPC growth and accelerating remyelination. Similarly, Spassky *et al*.^61^ provided evidence from embryonic models that class 3 semaphorins, such as Sema3A and Sema3F, together with netrin-1, guide OPC migration by exerting repulsive or attractive chemotactic effects. Although their study focused on the embryonic optic nerve, the same family of guidance cues is upregulated in the injured adult CNS, where they may contribute to the limited migration of OPCs by reinforcing localized retention. Thus, while vessel-derived signals influence adult OPC behaviour and differentiation, their migration to lesions is often a localized affair, heavily influenced by the inhibitory nature of the surrounding parenchymal ECM. Modulating this restrictive microenvironment is a key therapeutic goal.

As previously discussed, the perivascular niche also plays a crucial, non-migratory role, with pericytes depositing factors like laminin-α2 to promote OPC differentiation rather than their movement.^2^ Thus, the migration of pOPCs is ultimately a localized event, governed by a complex interplay of permissive and inhibitory signals within the microenvironment, the key components of which are summarized in Table 1.

**Table 1:** Summary of key signalling pathways in neural stem cell and oligodendrocyte precursor cell migration

Signalling pathway	Role in migration and remyelination	General role in CNS	Reference
Chemokine gradients (SDF-1/CXCL12 and CXCR4)	Establishes the primary long-distance chemoattractant gradient guiding SVZ-derived NSCs and transdifferentiating neuroblasts from their niche to lesion site.	Coordinates CNS response to injury by directing the homing of endogenous stem/progenitor cells to damaged areas.	Imitola *et al.*^47^
Wnt signalling pathway	Exerts dual control in the oligovascular niche; can either promote or stall OPC maturation. Dysregulated Wnt signalling can trap precursors in a perivascular, non-differentiating state.	Regulates stem cell fate decisions, maintains vascular integrity, and orchestrates tissue patterning during development and repair.	Azim *et al*.,^32^ Niu *et al*.^66^
Sonic hedgehog (Shh)/Gli signalling	Critically regulates the mobilization and oligodendrogenic fate of SVZ-NSCs. Inhibition of the downstream effector Gli1 enhances the accumulation of NSC-derived precursors at lesion sites.	A master regulator of developmental patterning and cell fate specification. In adults, it modulates the activity of resident stem cell populations.	Samanta *et al*.,^27,29^ Radecki *et al*.^28^
Bone morphogenetic protein (BMP) pathway	A major inhibitory pathway that potently blocks OPC differentiation, trapping precursors at the lesion. Can be aberrantly activated by blood-borne factors like fibrinogen after BBB breakdown.	Maintains homeostasis by tightly balancing cell proliferation and differentiation; prevents excessive or inappropriate cell maturation in development and repair.	Petersen *et al*.^26,67^
Cytokine/chemokine networks (IL-17 and CXCL5)	Components of the inflammatory milieu within the oligovascular niche that mediate communication between endothelial cells and precursors, influencing their recruitment to sites of white matter injury.	Modulates neuroinflammatory responses, acting as a bridge between the immune system and CNS-resident cells to coordinate tissue repair.	Xiao *et al*.^56^
Angiocrine factors (TGFβ1 and Ang1)	Pro-reparative signals (e.g. via Ang1-Tie2-TGFβ1 crosstalk) released by endothelial cells that help create a permissive niche, supporting precursor migration and subsequent differentiation.	Facilitates direct communication between the vasculature and neural cells, coupling vascular remodelling with parenchymal repair processes.	De La Fuente *et al*.,^2^ Su *et al*.^53^
ECM and environmental cues	Provides a complex mixture of permissive and inhibitory signals. Laminin-α2 deposited by pericytes promotes pOPC ‘differentiation’, not migration. CSPGs from reactive astrocytes create a physical and biochemical barrier that inhibits pOPC migration and process extension.	Forms the structural and biochemical scaffold of the CNS. The composition of the ECM dictates cell adhesion, motility, and differentiation, and is profoundly altered after injury.	De La Fuente *et al*.,^2^ Ghorbani *et al*.,^59^ Keough *et al*.^60^
Semaphorins	Act as critical guidance molecules. In development, astrocyte-derived Sema3a/6a actively mediate OPC detachment from vessels, a prerequisite for their terminal differentiation and myelination.	A diverse family of repulsive and attractive cues that govern axon guidance, cell positioning, and the precise formation of neural circuits	Su *et al*.,^53^ Spassky *et al*.^61^

Ang1 = angiopoietin-1; BBB = blood–brain barrier; BMP = bone morphogenetic protein; CSPG = chondroitin sulfate proteoglycan; CXCL5 = C-X-C motif chemokine ligand-5; CXCR4 = C-X-C chemokine receptor-4; ECM = extracellular matrix; Gli = glioma-associated oncogene transcription factors; IL = interleukin; Lama2 = laminin-α2; NSC = neural stem cell; OB = olfactory bulb; OPC = oligodendrocyte precursor cell; PlxnA3 = plexin-A3; RMS = rostral migratory stream; SDF-1 = stromal cell-derived factor-1, CXCL12; Sema3A/3F/6A/6B = semaphorins 3A/3F/6A/6B; Shh = sonic hedgehog; SVZ = subventricular zone; TGFβ = transforming growth factor-β; Wnt = Wingless/Int-1.

## Age-dependent barriers to remyelination in pOPC and SVZ niches

Although direct, head-to-head studies of how ageing alters both pOPC and SVZ-derived migration are limited, convergent primary evidence shows that ageing impairs the motility of these myelin precursors. Chari *et al*.^62^ showed that, after heterochronic grafts of age-mismatched adult OPCs (i.e. donor and host of different ages), old OPCs remained slow in young hosts while young OPCs remained fast in old hosts—demonstrating a cell-intrinsic motility deficit. Beyond cell-intrinsic changes, biophysical ageing of the niche impedes movement. OPCs migrate most efficiently on substrates within a stiffness range of ∼0.4–0.7 kPa, with motility declining outside this window.^63^ Age-related ECM remodelling stiffens the CNS environment, and this increased rigidity—sensed via piezo-type mechanosensitive ion channel component 1 (PIEZO1)- plausibly shifts OPCs off their migratory optimum, hindering recruitment.^64^ In addition to pOPC, ageing disrupts the SVZ ‘migration apparatus’ itself: 3D whole-mount reconstructions show neuroblast chains become sparser and less linear with age (i.e. degraded chain organization), and this is accompanied by sex-specific vascular remodelling in the niche. In aged males, vessels are denser and more tortuous; in aged females, they are fewer but larger and less tortuous. The spatial coupling between chains and vessels also shifts with age—chains lie closer to vessels in aged males but farther in aged females—indicating altered vessel-based guidance. Together, these structural changes impair long-range guidance of SVZ-derived cells, independent of proliferation.^65^

## A unifying model of precursor cell response in demyelination

The reparative response to demyelination can be conceptualized as a multi-phasic process, integrating the disparate actions of local and distal precursor pools within a dynamically evolving microenvironment. This model synthesizes the interplay between vessel-associated and vessel-independent migration, juxtaposing the rapid but spatially restricted response of parenchymal OPCs with the long-range, adaptive migration of progenitors from the SVZ. The process is initiated by the primary demyelinating insult, which frequently compromises the blood–brain barrier (BBB). This vascular breach precipitates the influx of blood-borne molecules, notably fibrinogen, which functions as a potent negative regulator of remyelination by aberrantly activating the BMP signalling pathway in OPCs and arresting their maturation.^26^ In this acute phase, resident pOPCs are the first responders; they rapidly proliferate at the lesion border, yet their migratory capacity is markedly restricted, limiting their contribution to the immediate periphery. Their movement, while modest, appears to use the local, albeit damaged, vasculature as a basic scaffold.

In parallel, injury signals originating from the lesion reach the SVZ, mobilizing a secondary wave of progenitors. This cohort includes not only NSCs but also committed neuroblasts that are induced to transdifferentiate. Recruited along potent chemokine corridors defined primarily by the SDF-1/CXCR4 axis, these cells begin a long-distance migration.^47^ They exhibit considerable migratory plasticity, capable of both leveraging reactive vasculature as a physical scaffold and navigating the brain parenchyma directly. Once released from the RMS, these cells travel via integrin-dependent mechanisms, demonstrating a capacity to adapt their migratory strategy to the available substrate.

**Figure 1:**
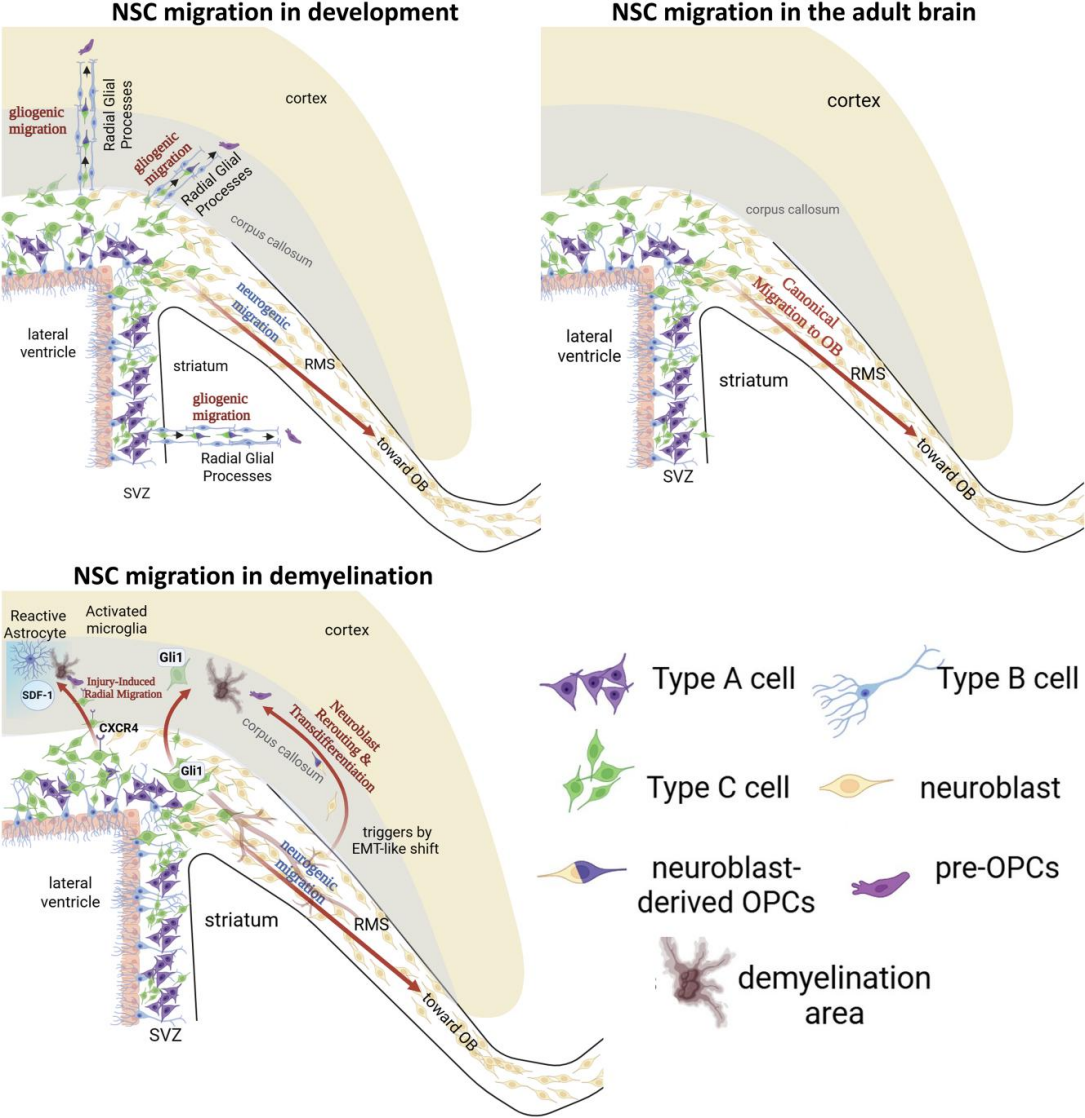
**Divergent migratory paradigms of SVZ-derived progenitors in development, adulthood and demyelination.** A schematic illustration of the distinct migratory routes and fates of cells originating from the subventricular zone (SVZ) across three different states. *Top left*: Development. During postnatal development, the SVZ orchestrates two primary migratory events. The tangential migration of neuroblasts occurs along the rostral migratory stream (RMS) toward the olfactory bulb (OB). Concurrently, a massive wave of glial progenitors undergoes vessel-independent radial migration, moving outwards along radial glial processes to populate the striatum, corpus callosum and cortex for subsequent gliogenesis. *Top right*: Adult brain. In the healthy adult brain, SVZ-derived migration is predominantly restricted to the canonical RMS pathway, where a continuous stream of neuroblasts replenishes interneurons in the olfactory bulb. *Bottom left*: Demyelination. In response to a demyelinating lesion, this homeostatic process is dynamically altered. A subset of SVZ-derived progenitors bypasses the RMS to undergo injury-induced radial migration toward the adjacent damaged area. Simultaneously, neuroblasts already in transit within the RMS can be rerouted. Triggered by lesion-derived signals, these cells undergo an epithelial-to-mesenchymal-like transition, exit the RMS and navigate toward the lesion, ultimately transdifferentiating into myelin-forming oligodendrocytes. This reparative response involves both vessel-dependent and vessel-independent migration guided by chemotactic gradients (e.g. SDF-1) released by reactive glia. NSC = neural stem cell; OPC = oligodendrocyte precursor cell. Created in BioRender. Ghorbani, S. (2025) https://BioRender.com/qbr5pf1.

**Figure 2:**
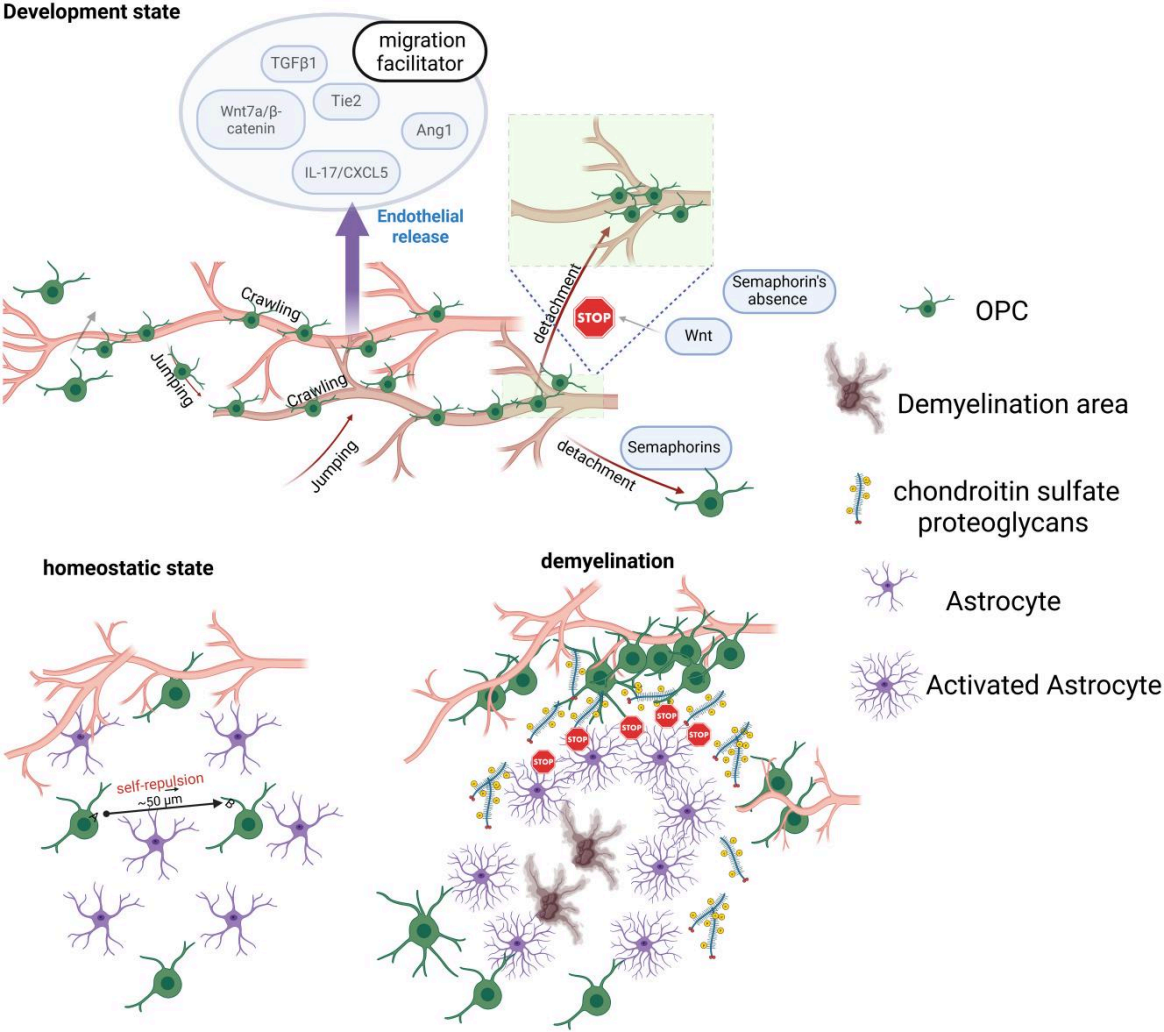
**A unifying model of oligodendrocyte precursor cell responses in homeostasis and demyelination.** This figure contrasts the migratory behaviours of resident parenchymal oligodendrocyte precursor cells (pOPCs) in healthy and pathological conditions. *Top*: Development state. During development, a sub-population of OPCs uses the nascent vasculature as a primary scaffold, migrating via ‘crawling’ and ‘jumping’ motions. Their movement is supported by endothelial-released migration facilitators (e.g. Wnt7a, Ang1). Critically, detachment from the vessel to allow for terminal differentiation is an active process mediated by repulsive cues like semaphorins; in the absence of such signals, OPCs remain associated with the vasculature. *Bottom left*: Homeostatic state. In the healthy adult CNS, pOPCs are quiescent and organized in non-overlapping domains maintained by self-repulsion and contact inhibition, ensuring the parenchyma is evenly tiled. Their processes continuously survey a defined territory of approximately 50 µm. *Bottom right*: Demyelination. Following a demyelinating insult, pOPCs become activated and proliferate at the lesion border. However, their long-range migration is severely impeded by a hostile microenvironment characterized by a dense glial scar rich in inhibitory chondroitin sulfate proteoglycans (CSPGs) secreted by reactive astrocytes. This creates a biochemical and physical barrier that arrests pOPC migration, preventing them from reaching the core of the lesion and effectively limiting their contribution to an abortive, peripheral repair attempt. Created in BioRender. Ghorbani, S. (2025) https://BioRender.com/l2mz8pn.

As these SVZ-derived progenitors meet with local pOPCs at the lesion site, the functionality of the ‘oligovascular niche’ becomes the decisive factor in determining the outcome. In resolving lesions, a collaboration between endothelial cells, pericytes, and astrocytes establishes a pro-remyelinating milieu. Pericytes, for instance, deposit laminin-α2 into the vascular basement membrane to promote pOPC differentiation, while astrocytes mediate the crucial detachment of precursors from vessels, a prerequisite for myelination.^2,53^ However, in the context of chronic inflammation and severe vascular damage, this supportive niche deteriorates. Precursors become arrested in a perivascular state, their differentiation blocked by sustained inhibitory signals from the BMP and Wnt pathways.^66,67^ The surrounding ECM becomes a non-permissive biochemical landscape. While this environment is rich in well-characterized inhibitors like CSPGs, it contains a much wider array of inhibitory molecules that impede cell migration and process outgrowth, as we have comprehensively reviewed.^59^ It is in this dysfunctional environment that the competition and compensation between the two precursor pools are most critical. This raises a critical unanswered question: is the inhibitory environment of the lesion less hostile to incoming SVZ-derived progenitors? In the absence of direct comparative studies, the model proposed in this review posits that their advantage lies not in an inherent resistance but in their recruitment via an entirely separate, long-range compensatory mechanism that is activated when the local pOPC response is impaired or proves insufficient.^37^ Ultimately, fostering a pro-reparative environment—a central goal of regenerative medicine—is a multifaceted process. This includes, among other factors, restoring BBB integrity to prevent the influx of inhibitory blood-derived molecules^26^ and resolving chronic inflammation to dismantle the inhibitory glial scar.^68^ This unifying model, which contrasts the distinct contributions of the local pOPC and distal SVZ-derived precursor pools (summarized in Table 2), proposes that effective remyelination is the outcome of this dynamic interplay, conceptually illustrated in Figs 1 and 2.

**Table 2:** Summary of neural stem cell and oligodendrocyte precursor cell migration mechanisms

Context/stage	Primary cell types	Migration pathways	Key guidance mechanisms	Central signalling pathways	Microenvironmental influences
Embryonic/postnatal development	SVZ-derived glial progenitors and ventrally-derived OPCs	Vessel-independent (dominant): radial egression radial glia from SVZ into corpus callosum and cortex. Vessel-dependent: a subpopulation of OPCs dispersal via ‘crawling’ and ‘jumping’.	Radial glial scaffolds are primary; vessels secondary, for SVZ-derived progenitors. Astrocyte-derived Sema3a/6a mediates OPC detachment from vessels; OPC-derived Sema6a/6b repels interneurons.	Signalling gradients (e.g. Cxcl12) provide general chemoattraction that can be locally overridden by repulsive cues. Transcriptional programmes temporally regulate the output of lineage-restricted glial progenitors.	Organized radial glial architecture provides clear, non-vascular migratory routes. Astrocyte endfeet physically displace OPCs from vessels to facilitate differentiation.
Adult brain (homeostatic RMS)	NSCs in the SVZ Migratory neuroblasts (type A cells)	Highly restricted tangential chain migration along the RMS to the olfactory bulb.	PSA-NCAM: lowers homophilic adhesion, permitting fluidic cell movement within chains. Glial tunnels: specialized astrocytes ensheath migrating chains, forming conduits.	Chemoattractants (SDF-1, BDNF) regulate the pace and directionality of migration within the RMS. Neurotransmitters (GABA) modulate migratory speed and synchrony of neuroblasts.	Mature vascular niche exists alongside the RMS. Adult ECM is more restrictive, limiting migration to the established stream.
Adult demyelination: SVZ response	SVZ-derived NSCs and their progeny Transdifferentiating neuroblasts	Vessel-dependent (adaptive): Co-opting of existing/newly formed reactive vasculature as primary scaffold. Vessel-independent: chain migration along non-stereotypical parenchymal routes.	Vascular scaffolds give physical guidance tracks. Integrins/focal adhesions provide traction for movement along vasculature and ECM. EMT-like shift (Zeb1/2, Scratch1) dismantles neuroblast chain adhesion to exit RMS.	SDF-1/CXCR4 gradient from lesion guides long-distance migration. Transcriptional reprogramming induces a transient oligodendroglial state without pluripotency.	Inflammatory/lesion cues (e.g. from reactive astrocytes) activate and mobilize NSCs. Demyelination signals dismantle the canonical RMS framework.
Adult demyelination: pOPC response	pOPCs	Severely constrained, short-range migration. Cell somata exhibit minimal displacement (avg. 2.1 µm/day). Local territory is repopulated via proliferation and invasion from immediate neighbours (∼50 µm).	Self-repulsion and contact inhibition: maintains tiled, non-overlapping pOPC domains and drives local cell replacement. Perivascular association: pOPCs are often near vessels, but this is a niche for differentiation, not long-range migration.	Pericyte-derived Laminin-α2 (Lama2) promotes pOPC differentiation and survival, not migration. Inhibitory signalling from CSPGs in the glial scar can impede process extension. Inhibitory signalling from aberrant developmental pathways (BMP, Wnt): potently stall differentiation.	Dense, inhibitory ECM from astrogliosis (e.g. CSPGs, fibrin, tenascins) biochemically and physically hinders motility. Competition from newly arrived SVZ-derived OPCs can occur if pOPC function is impaired.

BDNF = brain-derived neurotrophic factor; BMP = bone morphogenetic protein; CSPGs = chondroitin sulfate proteoglycans; CXCR4 = C-X-C chemokine receptor-4; ECM = extracellular matrix; EMT = epithelial-to-mesenchymal-like transition; GABA = γ-aminobutyric acid; Lama2 = laminin-α2; NSC = neural stem cell; OPC = oligodendrocyte precursor cell; pOPC = parenchymal OPC; PSA-NCAM = polysialylated neural cell adhesion molecule; RMS = rostral migratory stream; Scratch1 = snail family transcriptional repressor-1.; SDF-1/CXCL12 = stromal cell-derived factor-1; SVZ = subventricular zone; Wnt = Wingless/Int-1; Zeb1/2 = zinc-finger E-box-binding homeobox-1/2.

Incorporating species differences, our model predicts that human remyelination relies predominantly on pOPCs, with more limited and context-dependent recruitment from the adult SVZ compared with rodents. Adult human SVZ neurogenesis and long-range migration are markedly attenuated relative to rodents, with human oligodendroglial support shifting toward local, parenchymal repair; consistent with broader evidence that adult human migratory corridors are restricted and contentious compared with rodent paradigms.^69^ In parallel, single nucleus RNA sequencing of human MS white matter shows that oligodendrocyte lineage states broadly map onto the corresponding mouse progenitor and mature states, indicating substantial conservation; nonetheless, human tissues display disease-associated programmes and shifts in state proportions, so rodent-derived targets should be calibrated in human contexts.^70^ Accordingly, we suggest that translational priorities should be OPC-centric by mitigating disease-associated extrinsic brakes and removing ECM barriers to migration and process extension, while normalizing mechanical cues that degrade OPC motility with age (stiffness).

## Conclusion

The journey of NSCs and OPCs to sites of demyelination is a testament to the brain's intrinsic, albeit often limited, capacity for self-repair. Our understanding has evolved significantly, moving beyond a simplistic model of migration along vascular ‘highways’ to a more sophisticated understanding of a dynamic interplay between vessel-dependent and vessel-independent guidance. This review has synthesized evidence demonstrating that while the oligovascular niche—comprising endothelial cells, pericytes, and astrocytes—is a critical signalling hub, both SVZ-derived NSCs and parenchymal OPCs are guided by a complex and context-dependent integration of vascular scaffolds, chemotactic gradients, and the local ECM.

A central theme emerging from the current body of literature is the distinct contribution of the two primary precursor pools. Endogenous pOPCs mount a rapid, local response, but their homeostatic, self-repulsive properties severely constrain their long-distance migration, rendering them effective only in the immediate lesion periphery. In contrast, the adult SVZ contains a more plastic population of progenitors—including transdifferentiating neuroblasts—that can traverse considerable distances by leveraging both vascular and parenchymal routes guided by potent chemokines like SDF-1. The success of remyelination often depends on a competition or compensation between these two populations, where the failure of the local pOPC response necessitates the successful recruitment of this long-range, SVZ-derived cohort.^37^

The ultimate efficacy of either precursor population is dictated by the lesion microenvironment. Chronic inflammation, BBB disruption, and reactive astrogliosis, along with other factors beyond the scope of this review, can transform the niche from supportive to inhibitory, stalling differentiation through the aberrant reactivation of developmental signalling pathways (such as BMP and Wnt), deposition of an inhibitory extracellular matrix, rich in inhibitors like CSPGs, creates a biochemical barrier that obstructs migration and process extension. Therefore, future therapeutic interventions must be multi-pronged, designed to modulate this complex and often dysfunctional ecosystem. Key strategies, directly informed by these mechanistic insights, should aim to: (i) enhance the recruitment and targeted migration of the plastic SVZ-derived progenitors; (ii) overcome the local, inhibitory barriers that restrict pOPC migration and process outgrowth; and (iii) resolve the differentiation arrest common to both cell types by rebalancing dysregulated signalling pathways within the oligovascular niche.

## Supplementary Material

awaf441_Supplementary_Data
