# Supplementary material for: Routes of precursors’ migration in remyelination
Source: Brain. 2025 Nov 20;149(4):1124–36. doi: 10.1093/brain/awaf441 (PMC13058462; doi:10.1093/brain/awaf441)
Supplement: awaf441_Supplementary_Data [file awaf441_supplementary_data.pdf]

Supplementary File

Routes of Precursors' Migration in Remyelination

Majid Ghareghani[1,2,3] & Samira Ghorbani[1,2,3*]

Supplementary Table 1: Summary of stimulatory cues that promote differentiation of adult SVZ-derived NSCs OPCs.

Type of Cue	Model & Experimental Approach	Key Findings	Reference
**Demyelination (injury) – environmental cue**	Mouse in vivo (corpus callosum demyelination lesion); fate-mapping of SVZ NSCs	Demyelinating injury stimulates SVZ NSCs to generate OPCs that migrate to lesions. OPC output from NSCs increased ~4-fold after lesion, indicating NSCs contribute to remyelination.	(Menn, Garcia-Verdugo et al. 2006)
**Wnt/β-catenin signaling – chemical/niche cue**	Mouse in vivo (postnatal SVZ); infusion of GSK3β inhibitor (activates Wnt signaling)	Pharmacological Wnt activation in the dorsal SVZ dramatically increased generation of oligodendrocyte precursors from NSCs. Wnt/β-catenin signaling in dorsal SVZ drives OPC and glutamatergic neuron specification.	(Azim, Fischer et al. 2014)
**Sonic Hedgehog (Shh) – niche morphogen**	Mouse in vivo (early postnatal SVZ); $Gli1^+$ NSC lineage tracing, Shh level manipulations	Identified a dorsal SVZ domain with high Shh signaling that produces many oligodendroglial lineage cells. The number of OPCs generated correlated with Shh signal levels, and blocking Shh reduced SVZ-derived oligodendrogenesis. Shh is required for robust OPC production in this region.	(Tong, Fuentealba et al. 2015)
**EGF/EGFR signaling – growth factor**	Mouse in vivo (postnatal); transgenic overexpression of human EGFR (CNP–hEGFR) and EGFR hypomorphic mutants; demyelination model	EGFR overexpression in SVZ progenitors expanded $NG2^+Mash1^+Olig2^+$ oligodendroglial progenitors, accelerated their migration to demyelinated lesions, and improved remyelination. EGFR-hypomorphic mice had impaired OPC production. Demonstrates EGFR signaling enhances NSC-derived OPC generation and myelin repair.	(Aguirre, Dupree et al. 2007)
**FGF/FGFR3 signaling – genetic activation**	Mouse in vivo (adult); inducible activation of FGFR3 in SVZ NSCs (transgenic); corpus callosum demyelination	Transiently increasing FGFR3 activity in adult NSCs redirected NSCs from neuron to oligodendroglial lineage during demyelination. Led to a surge in SVZ-derived OPC production and significantly improved oligodendrocyte regeneration and remyelination. Identifies FGF signaling as a potent target to bias NSCs toward OPC fate.	(Kang, Nguyen et al. 2019)
**Hepatoma-derived growth factor (HDGF; Growth factor) – extrinsic cue**	Mouse SVZ NSC culture (postnatal) and in vivo infusion (adult mice)	Exogenous HDGF protein enhanced oligodendrocyte genesis from SVZ neural precursors in vitro, without increasing neuron or astrocyte production. In vivo intracerebroventricular infusion of HDGF similarly increased OPC generation from the adult SVZ. Uncovered HDGF as a novel niche signal promoting NSC-to-OPC differentiation.	(Li, Dittmann et al. 2022)
**Hypothyroid condition – systemic/environmental cue**	Mouse in vivo (adult); transient induction of hypothyroidism during demyelination (anti-thyroid drug)	Transient adult hypothyroidism favored OPC lineage commitment of SVZ stem cells. Mice with reduced thyroid hormone had increased SVZ-derived OPC production and more robust remyelination of lesions. Indicates low thyroid hormone (an environmental hormone cue) stimulates NSCs to generate OPCs for repair.	(Remaud, Ortiz et al. 2017)
**Circadian/lesion signals – niche/injury cue**	Mouse in vivo; demyelination lesion sending signals to SVZ (circadian disruption); organotypic slice culture and in vivo analysis	Demyelinated lesions were found to secrete SFRP1/5 (Wnt inhibitors) in a circadian manner, which traveled to the SVZ and triggered NSCs to adopt oligodendrocyte lineage by downregulating the clock gene Bmal1. This inter-tissue signaling significantly increased oligodendrogenesis from NSCs and enhanced remyelination. Reveals that lesion environments can actively signal NSCs to become OPCs.	(Huang, Choi et al. 2020)
**Olig2 transcription factor – intrinsic/genetic cue**	Mouse in vivo (early postnatal SVZ); retroviral Olig2 overexpression and dominant-negative Olig2; fate analysis	The bHLH factor Olig2 was shown to be necessary and sufficient to direct SVZ progenitors toward oligodendrocyte (and astrocyte) fates and suppress neuronal differentiation. Constitutive Olig2 activity in NSCs prevented neurogenesis and induced oligodendroglial lineage entry. First demonstration that an intrinsic factor (Olig2) tilts the NSC differentiation program toward OPCs.	(Marshall, Novitch et al. 2005)

**Zfp488 transcription factor – intrinsic/genetic cue**	Mouse in vivo (adult; cuprizone demyelination); retroviral delivery of Zfp488 into SVZ NSCs	Overexpression of the oligodendrocyte-specific factor Zfp488 in adult SVZ NSCs robustly promoted their differentiation into oligodendrocyte lineage cells. Zfp488-transduced NSCs generated mature oligodendrocytes in vivo, leading to significantly improved remyelination and functional recovery after demyelination. Identifies Zfp488 as a powerful driver of NSC-to-OPC differentiation in the adult brain.	(Soundarapandian, Selvaraj et al. 2011)
**Forced OPC programming in human NSCs – genetic cue (transcription factor)**	Human ES/iPS-derived NSCs in vitro; lentiviral Zfp488 overexpression; transplantation into mice	Artificially expressing oligodendrocyte TF Zfp488 in human NSCs caused an exclusive commitment to the OPC lineage during differentiation. Treated hNSCs largely ceased neuron/astro differentiation and produced abundant OPCs and oligodendrocytes. When transplanted into shiverer (myelin-deficient) mice, the Zfp488-programmed hNSCs migrated to white matter, became pre-myelinating oligodendrocytes, and formed myelin in vivo. Demonstrates a translational strategy of genetically steering human NSCs to OPCs for therapy.	(Biswas, Chung et al. 2019)

Footnote: **Abbreviations:** BMAL1 = Brain and Muscle ARNT-Like 1 (core circadian transcription factor); DOI = Digital Object Identifier; EGF = Epidermal Growth Factor; EGFR = Epidermal Growth Factor Receptor; FGF = Fibroblast Growth Factor; FGFR3 = Fibroblast Growth Factor Receptor 3; GSK3β = Glycogen-Synthase-Kinase 3 beta; HDGF = Hepatoma-Derived Growth Factor; hNSC = human Neural Stem Cell; NSC = Neural Stem Cell; NMDA = N-Methyl-D-Aspartate (glutamate receptor subtype); OPC = Oligodendrocyte Progenitor Cell; Olig2 = Oligodendrocyte Transcription Factor 2; Shh = Sonic Hedgehog; SFRP = Secreted Frizzled-Related Protein; SVZ = Subventricular Zone; TF = Transcription Factor; Wnt = Wingless/Int-1 signaling pathway; Zfp488 = Zinc-Finger Protein 488.

References

Aguirre, A., J. L. Dupree, J. M. Mangin and V. Gallo (2007). "A functional role for EGFR signaling in myelination and remyelination." Nat Neurosci **10**(8): 990–1002.

Azim, K., B. Fischer, A. Hurtado-Chong, K. Draganova, C. Cantù, M. Zemke, L. Sommer, A. Butt and O. Raineteau (2014). "Persistent Wnt/β-catenin signaling determines dorsalization of the postnatal subventricular zone and neural stem cell specification into oligodendrocytes and glutamatergic neurons." Stem Cells **32**(5): 1301–1312.

Biswas, S., S. H. Chung, P. Jiang, S. Dehghan and W. Deng (2019). "Development of glial restricted human neural stem cells for oligodendrocyte differentiation in vitro and in vivo." Scientific Reports **9**(1): 9013.

Huang, S., M. H. Choi, H. Huang, X. Wang, Y. C. Chang and J. Y. Kim (2020). "Demyelination Regulates the Circadian Transcription Factor BMAL1 to Signal Adult Neural Stem Cells to Initiate Oligodendrogenesis." Cell Reports **33**(7).

Kang, W., K. C. Q. Nguyen and J. M. Hébert (2019). "Transient Redirection of SVZ Stem Cells to Oligodendrogenesis by FGFR3 Activation Promotes Remyelination." Stem Cell Reports **12**(6): 1223–1231.

Li, Y., N. L. Dittmann, A. Eve, S. Watson, M. M. A. de Almeida, T. Footz and A. Voronova (2022). "Hepatoma Derived Growth Factor Enhances Oligodendrocyte Genesis from Subventricular Zone Precursor Cells." ASN Neuro **14**: 17590914221086340.

Marshall, C. A., B. G. Novitch and J. E. Goldman (2005). "Olig2 directs astrocyte and oligodendrocyte formation in postnatal subventricular zone cells." J Neurosci **25**(32): 7289–7298.
Menn, B., J. M. Garcia-Verdugo, C. Yaschine, O. Gonzalez-Perez, D. Rowitch and A. Alvarez-Buylla (2006). "Origin of Oligodendrocytes in the Subventricular Zone of the Adult Brain." The Journal of Neuroscience **26**(30): 7907.

Remaud, S., F. C. Ortiz, M. Perret-Jeanneret, M. S. Aigrot, J. D. Gothié, C. Fekete, Z. Kvárta-Papp, B. Gereben, D. Langui, C. Lubetzki, M. C. Angulo, B. Zalc and B. Demeneix (2017). "Transient hypothyroidism favors oligodendrocyte generation providing functional remyelination in the adult mouse brain." Elife **6**.

Soundarapandian, M. M., V. Selvaraj, U. G. Lo, M. S. Golub, D. H. Feldman, D. E. Pleasure and W. Deng (2011). "Zfp488 promotes oligodendrocyte differentiation of neural progenitor cells in adult mice after demyelination." Scientific Reports **1**(1): 2.

Tong, C. K., L. C. Fuentealba, J. K. Shah, R. A. Lindquist, R. A. Ihrie, C. D. Guinto, J. L. Rodas-Rodriguez and A. Alvarez-Buylla (2015). "A Dorsal SHH-Dependent Domain in the V-SVZ Produces Large Numbers of Oligodendroglial Lineage Cells in the Postnatal Brain." Stem Cell Reports **5**(4): 461–470.
